# A comparative study on the validations of three cognitive screening tests in identifying subtle cognitive decline

**DOI:** 10.1186/s12883-020-01657-9

**Published:** 2020-03-05

**Authors:** Feng-Feng Pan, Lin Huang, Ke-liang Chen, Qian-hua Zhao, Qi-hao Guo

**Affiliations:** 1 Department of Gerontology, Shanghai Jiao Tong University Affiliated Sixth People’s Hospital, No. 600, Yi Shan Road, Shanghai, P. R. China; 2 Department of Neurology and Institute of Neurology, Huashan Hospital, Shanghai Medical College, Fudan University, Shanghai, China

**Keywords:** Subtle cognitive decline (SCD), Mild cognitive impairment (MCI), Alzheimer’s disease (AD), Memory and executive screening (MES), Mini-mental state examination (MMSE), Montreal cognitive assessment-Chinese version (MoCA-CV)

## Abstract

**Background:**

Subtle cognitive decline (SCD) may represent a very early stage of objective cognitive impairment before mild cognitive impairment (MCI), with less neuronal damage and more functional reservation. Detecting individuals with SCD is imperative for dementia prevention and treatment. In this study, we aimed to compare the validations of three cognitive screening tests, Mini-Mental State Examination (MMSE), Montreal Cognitive Assessment-Chinese Version (MoCA-CV), and Memory and Executive Screening (MES), in identifying subtle cognitive decline.

**Methods:**

A total of 407 individuals were recruited, including 147 cognitively normal controls (NC), 102 individuals with subtle cognitive decline (SCD) and 158 individuals with mild cognitive impairment (MCI) according to the operational neuropsychological criteria proposed by Jak and Bondi’s. All participants underwent standardized comprehensive neuropsychological tests and the three cognitive screening tests. Chi-square analysis was used to compare the cognitive performance among the groups of NC, SCD and MCI. Receiver operating characteristic (ROC) curves were used to evaluate the abilities of MMSE, MoCA-CV and MES in discriminating NC, SCD and MCI.

**Results:**

Compared with NC, SCD showed a significant decline only in the tests of memory, such as Auditory Verbal Learning Test (AVLT), Rey-Osterrieth Complex Figure Test (CFT) and Prospective Memory Test (PrM) (*P* < 0.01). However, MCI showed significant decline in all cognitive performances (*P* < 0.01). The scores of MMSE, MoCA-CV and MES all showed a progressive downward trend within the groups of NC, SCD and MCI (*P* < 0.001). In ROC Analyses for discriminating individuals with SCD from NC, the most appropriate MES cutoff was 84, with a sensitivity of 74.3%, a specificity of 60.8% and 0.738 for AUC (95%CI, 0.675–0.801). By contrast, MMSE and MOCA-CV had poor sensitivity (67.4 and 70.8%, respectively) and specificity (51.0 and 52.9%, respectively), and smaller AUCs (0.643 and 0.644, respectively) than the MES.

**Conclusion:**

As a screening test, MES is more efficacious in identifying SCD from normal controls than MMSE and MoCA-CV.

## Background

With the aging of the world population, about 40 million people have dementia worldwide, mostly older than 60 years, and this figure will amount to 115 million in 2050 [1]. Currently, there is no modifying-course therapy for the most common cause of dementia, Alzheimer’s disease (AD).

Mild cognitive impairment (MCI) is considered as a transitional phase between normal cognitive aging and dementia. However, neuronal loss and cognitive impairment may have progressively occurred at this stage [2]. As a result, detecting individuals with a stage of less neuronal damage and more functional reservation is imperative for dementia prevention and treatment [3, 4]. Subtle cognitive decline (SCD), one of the markers which were used to define the preclinical stages of AD according to the National Institute on Aging and Alzheimer’s Association (NIA-AA) [5], may represent very subtle neurobehavioral changes occurred years before meeting the criteria for MCI. Moreover, education related cognitive reserve plays an important role in cognitive decline before and after the clinical diagnosis of AD [6]. Prior to the clinical diagnosis of dementia, cognitive reserve appears to exert a protective effect on cognitive decline [7]. Even in the middle-aged population, associations of global cognition and neuroimaging markers for neurodegeneration were influenced by cognitive reserve [8]. Thus, cognitive reserve may conceal the prominent symptoms though the brain pathology is already quite advanced. With the improvement of education level in the world today, increasing cognitive reserve makes individuals with certain pathological changes more likely to appear as SCD than prominent cognitive impairment.

A battery of standardized neuropsychological tests put forward by Jak and Bondi showed good sensitivity and reliability in diagnosing MCI, demonstrated a significant association with AD biomarkers and progression to dementia [9, 10]. This comprehensive neuropsychological method was also used in the operational definition of SCD within preclinical AD populations [11]. Six neuropsychological indexes are examined within this method to define the MCI and SCD individuals: Auditory Verbal Learning Test (AVLT) [12] 30-min delayed free recall and AVLT recognition for measurements of memory; Animal Verbal Fluency Test (AFT) [13] and Boston Naming Test (BNT) [14] for measurements of language; Trail Making Test Part A (TMT-A) and TMT part B (TMT-B) [15] for measurements of attention/executive function. Although these classical tests have good reliability and validity in the assessment of different cognitive domains, many factors still limit the application of these scales altogether, especially in cognitive screening among community population. First, these standardized neuropsychological tests need to be administered by trained raters but not general healthcare providers, while most Chinese hospitals do not have professional rater by now. Second, illiterate and low-educated people remain a significant proportion in the elderly population of China, and scales like AVLT are prone to be false-positive in this population. In addition, because of China’s vast territory, there are huge differences between the areas of south and north, urban and rural. It’s difficult to establish cut-off values of these scales locally. Therefore, what we need imperatively is a brief test without a necessity for strictly training among raters, widely acceptable for illiterate and low-educated people, and also with high sensitivity and specificity in detecting SCD.

Currently, there are many screening tests for MCI, such as Addenbrooke’s Cognitive Examination Revised (ACE-R) [16] and the Montreal Cognitive Assessment (MoCA) [17]. Besides, Memory and Executive Screening (MES), a cognition test with seven brief tasks, was confirmed as a valid and easily administered screening tool for MCI [18]. In this test, a sentence is remembered three times and delay recalled two times, which reflects instant and delayed memory. A category fluency subtest about enumerating things in the kitchen is used to reflect language function. The other three subtests including sequential movement task, conflicting instructions task and Go/No-go task, are all used to reflect executive function. Although these same screening tests are equally effective in early identification of MCI and AD, each test showed different sensitivity and specificity in identifying different degrees of cognitive impairment [19]. Even the same screening test showed different evaluation criteria in different population [20]. For SCD screening, the first thing we thought is to verify the effectiveness of MCI and AD screening tests in the identification of SCD. This study aimed to compare the validations of Mini-Mental State Examination (MMSE) [21], MoCA-CV and MES in identifying SCD and determine the corresponding optimal cutoff point.

## Methods

### Participants

A total of 407 individuals were recruited, including 147 cognitively normal controls (NC), 102 individuals with subtle cognitive decline (SCD) and 158 individuals with mild cognitive impairment (MCI). The participants with SCD and MCI were recruited from the Memory Clinic, Huashan Hospital, Shanghai, China, from 1/1/2016/ to 1/1/2018. Inclusion criteria were: aged more than 50 years old; educated more than 6 years; normal vision and hearing to complete cognitive tests; no history of alcoholism and drug abuse, or head trauma; Clinical Dementia Rating (CDR) [22] score ≤ 0.5; preserved basic activities of daily living (ADL) [23]; Hamilton depression rating scale (17-item) [24] score ≤ 12; and did not meet the diagnostic criteria of dementia based on the recommendations from the National Institute on Aging-Alzheimer’s Association (NIA-AA) workgroups [25]. Normal controls were recruited from Jinshan Community, Shanghai, China. Besides the common inclusion criteria, all the controls had no significant impairment in cognitive functions, preserved in activities of daily living, had no memory complaints verified by informants, CDR score = 0, Hamilton depression rating scale (17-item) score ≤ 12. Relevant laboratory screening and cranial MRI scanning were carried out in all participants. Individuals with significant abnormalities in folic acid, vitamin B12, thyroid function, rapid plasma regain (RPR), treponema pallidum particle agglutination (TPPA), or other serious neuropsychiatric diseases were excluded.

### Measures

Except for the cognition screening tests of MMSE, MoCA-Chinese Version (MoCA-CV) [26] and MES, all participants underwent extensive neuropsychological tests of memory, language, attention, executive function, and visuospatial ability. The tests included: Auditory Verbal Learning Test (AVLT), Prospective Memory Test (PrM) [27], Boston Naming Test (BNT), Animal Verbal Fluency Test (AFT), Symbol Digit Modalities Test (SDMT) [28], Digit Ordering Test (DOT) [29], Trail Making Test-A and B (TMT-A, TMT-B), Stroop Color-Word Test (SCWT) [30], and Rey-Osterrieth Complex Figure Test (CFT) [31]. Functional Activities Questionnaire (FAQ) [32] was also used to assess functional capacity based on the reports of informants. All neuropsychological assessments were carried out by trained raters who were blind to diagnosis.

As previously mentioned, in our non-demented participants, six neuropsychological indexes proposed by Jak and Bondi were used to operationalize the diagnosis of MCI and SCD [10, 11]. Individuals were diagnosed with MCI if they had any of the following criteria: (1) have impaired scores (defined as > 1 standard deviation (SD) below age-corrected normative mean) on two of the six neuropsychological measures in the same cognitive domain (either memory, language, or attention/executive function); (2) have impaired scores (defined as > 1 SD below age-corrected normative mean) in each of the three cognitive domains sampled; (3) Functional Assessment Questionnaire (FAQ) score ≥ 9. Similarly, Individuals were considered to have SCD if they had any of the following criteria: (1) have impaired scores (defined as> 1 SD below the age-corrected normative mean) on two of the six neuropsychological measures in different cognitive domains; (2) have an FAQ score of 6–8.

### Statistical analyses

Chi-square analysis and one-way analysis of variance were used to assess possible group differences between the three groups (SCD, AD, NC) in demographic characteristics and cognitive test performance. Post hoc pairwise between-group comparisons were assessed using the least significant difference test. Receiver operating characteristic (ROC) curves were used to determine the ability of MMSE, MoCA-CV and MES in discriminating among participants with normal cognition, SCD and MCI. The area under the ROC curve (AUC) was used to compare the diagnostic performance of the MMSE, MoCA-CV and MES. The level of significance was set at α = 0.05. All analyses were conducted using SPSS version 19.0 (IBM Corp., Armonk, NY).

## Results

### Demographics and standardized neuropsychological tests among NC, SCD, MCI

A total of 407 participants were included in our analysis, including 147 cognitively normal controls (NC), 102 individuals with SCD and 158 individuals with MCI according to the criteria of Jak and Bondi’s [13, 21]. Demographic features and the standardized neuropsychological test scores of each group are presented in Table 1. No significant difference was found in age and sex among the three groups. The years of education were a little but significantly higher in cognitively normal controls than the individuals of SCD and MCI (*p* = 0.004).

**Table 1:** Demographics and standardized neuropsychological tests for NC, SCD, MCI

Index	NC (*n* = 147)	SCD (*n* = 102)	MCI (*n* = 158)	F(*P* value)
*Demographics*				
Age (years)	66.3 ± 8.3	66.5 ± 9.5	66.6 ± 8.7	0.048 (0.953)
Education (years)	13.2 ± 2.9	12.1 ± 3.1#	12.2 ± 3.1*	5.684 (0.004)
Sex(M:F)	64:83	40:62	83:75	4.983 (0.084)
*Neuropsychological*				
AVLT delayed recall	6.1 ± 1.9	3.5 ± 1.1##	0.8 ± 1.1**††	468.601(< 0.001)
AVLT recognition	21.5 ± 2.2	19.7 ± 2.4##	16.6 ± 3.8**††	101.097(< 0.001)
Rey CFT copy	33.3 ± 3.1	32.2 ± 5.4	30.7 ± 6.1**	10.344(< 0.001)
Rey CFT recall	16.0 ± 6.1	12.9 ± 6.7##	8.2 ± 6.2**††	57.607(< 0.001)
TMT-A	53.9 ± 24.5	61.8 ± 37.0	76.6 ± 46.3**††	14.094(< 0.001)
TMT-B	132.5 ± 59.1	149.2 ± 62.7	194.8 ± 94.8**††	26.189(< 0.001)
SCWT-CR	46.0 ± 5.1	44.6 ± 6.0	40.3 ± 9.9**††	22.848(< 0.001)
SCWT-CT	76.6 ± 20.8	85.3 ± 24.6	99.4 ± 48.7**††	18.150(< 0.001)
BNT	24.2 ± 4.1	23.4 ± 3.5	21.5 ± 4.2**††	17.217(< 0.001)
AFT	16.9 ± 4.3	16.0 ± 4.1	13.9 ± 4.1**††	14.343(< 0.001)
SDMT	38.9 ± 11.8	36.0 ± 12.5	29.5 ± 11.1**††	24.839(< 0.001)
PrM	14.2 ± 4.2	12.0 ± 4.8##	7.2 ± 5.6**††	73.337(< 0.001)
DOT	4.9 ± 1.5	4.8 ± 1.0	3.9 ± 1.4**††	23.177(< 0.001)

Comparations between NC and SCD are marked behind SCD, # for *P* < 0.05, ## for *P* < 0.01

Comparations between NC and MCI are marked behind MCI, * for *P* < 0.05, ** for *P* < 0.01

Comparations between SCD and MCI are marked behind MCI, † for *P* < 0.05, †† for *P* < 0.01

*AVLT* Auditory Verbal Learning Test, *Rey CFT* Rey-Osterrieth Complex Figure Test, *TMT-A*, *TMT-B* Trail Making Test Part A and B, *SCWT-CR/CT* Stroop Color Word Test-Card C right/Card C time, *BNT* Boston Naming Test, *SDMT* Symbol Digit Modalities Test, *PrM* Prospective Memory Test, *DOT* Digit Ordering Test

Compared with the group of NC, SCD showed significant decline only in memory tests, such as AVLT 30-min delayed free recall, AVLT recognition, Rey-Osterrieth CFT recall and PrM test. There was no significant difference in other cognitive performances between SCD and NC. However, the MCI group showed significant decline in all cognitive performances in comparison with the NC group, such as AVLT, Rey CFT recall and PrM tests for memory, BNT and AFT tests for language, SDMT, TMT-A, TMT-B and SCWT tests for attention and executive, DOT for verbal working memory, Rey CFT copy test for visuospatial ability. Even compared with the group of SCD, the MCI group showed obvious decline in most cognitive tests except for Rey CFT copy test.

### Total scores of screening tests among NC, SCD, MCI

In our study, MMSE, MoCA-CV, MES were used as screening tests for discriminating NC, SCD, MCI. Total scores of the three screening tests in each group are presented in Table 2. The scores of MMSE, MoCA-CV and MES all showed a progressive downward trend within the groups of NC, SCD and MCI (*P* < 0.001). Pairwise comparison showed that, between the groups of NC and SCD, the average MMSE score distance was less than 1 point, and the average MoCA-CV score distance was only 1.5 points. However, the average MES score distance between NC and SCD was more than 8 points. Similar results were obtained between the groups of SCD and MCI.

**Table 2:** Comparison of three screening tests for NC, SCD, MCI

Index	NC (*n* = 147)	SCD (*n* = 102)	MCI (*n* = 158)	F(*P* value)
MMSE	28.1 ± 1.7	27.3 ± 1.7##	26.2 ± 1.9**††	40.619(< 0.001)
MOCA-CV	24.6 ± 3.0	23.1 ± 3.0##	20.2 ± 3.3**††	76.830(< 0.001)
MES	87.7 ± 10.5	79.5 ± 9.4##	70.6 ± 11.7**††	95.495(< 0.001)

Comparations between NC and SCD are marked behind SCD, ## for *P* < 0.01

Comparations between NC and MCI are marked behind MCI, ** for *P* < 0.01

Comparations between SCD and MCI are marked behind MCI, †† for P < 0.01

*MMSE* Mini-Mental State Examination, *MoCA-CV* Montreal Cognitive Assessment-Chinese Version, *MES* Memory and Executive Screening

### ROC analyses of MMSE, MoCA-CV and MES in discrimination among NC, SCD and MCI

**Fig. 1:**
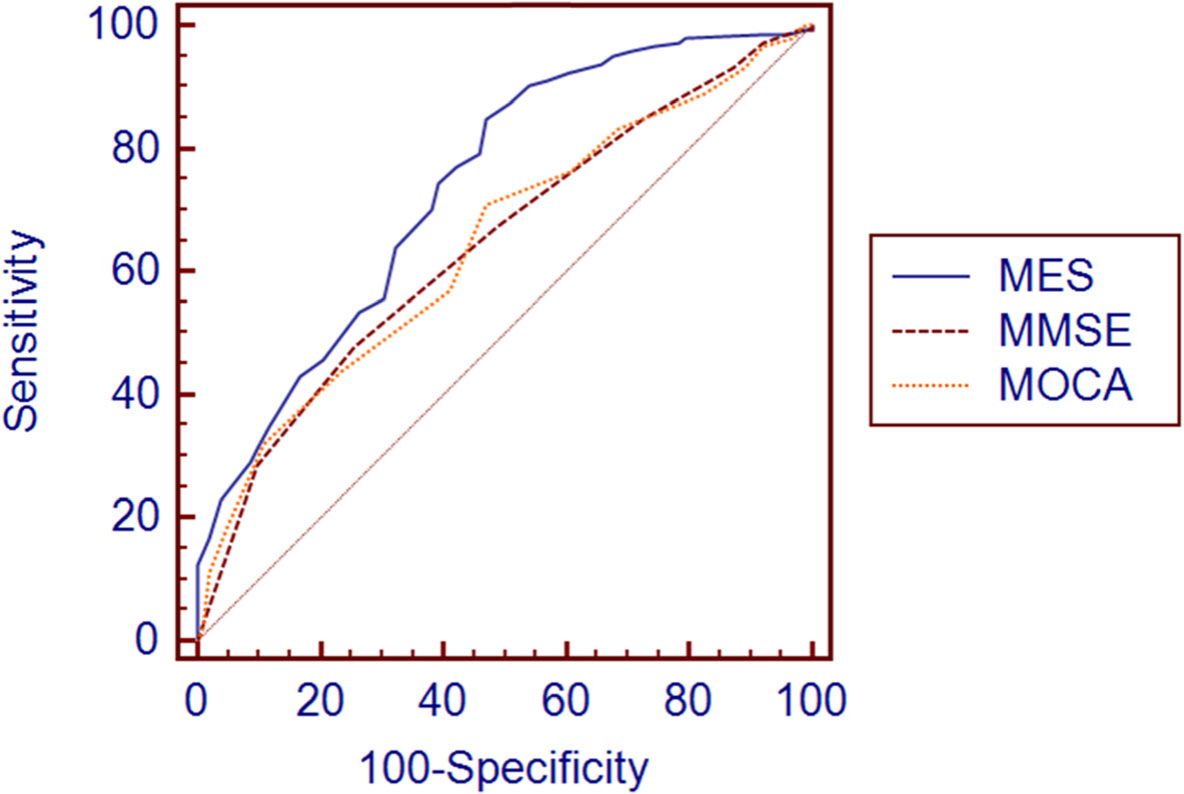
ROC curve for SCD vs NC

**Fig. 2:**
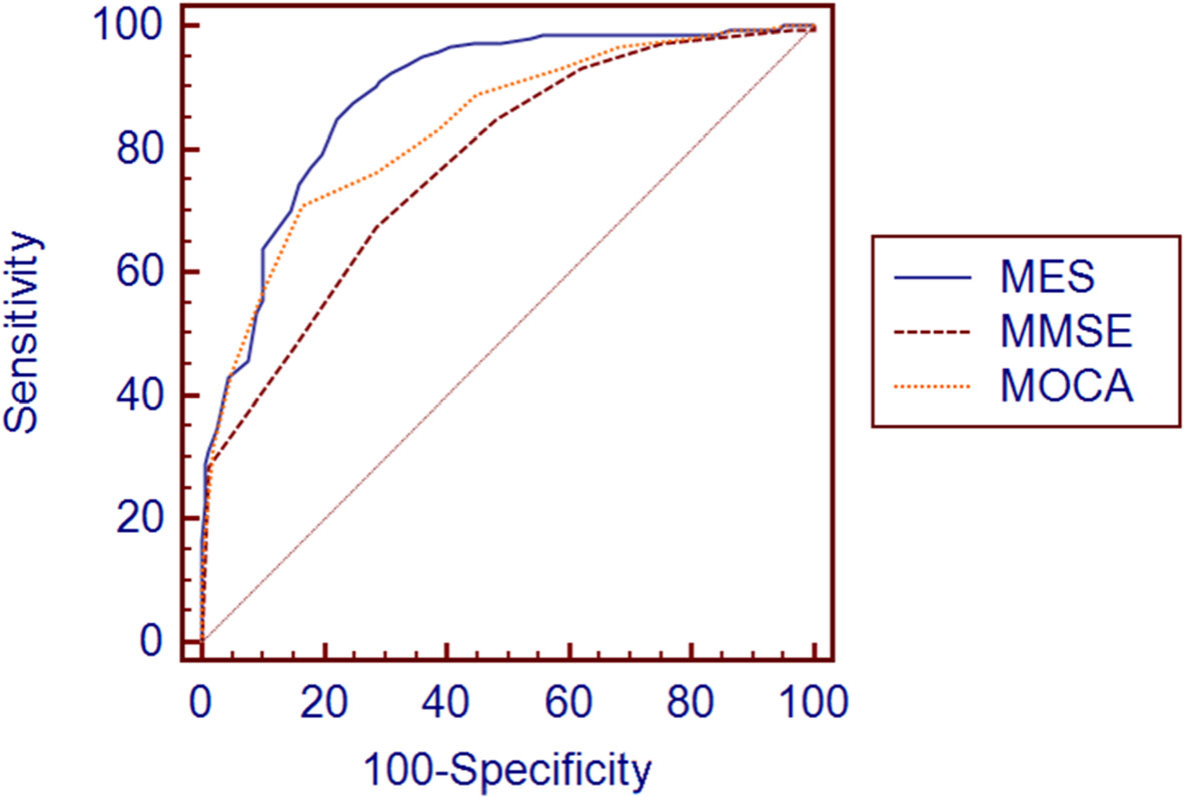
ROC curve for MCI vs NC

**Fig. 3:**
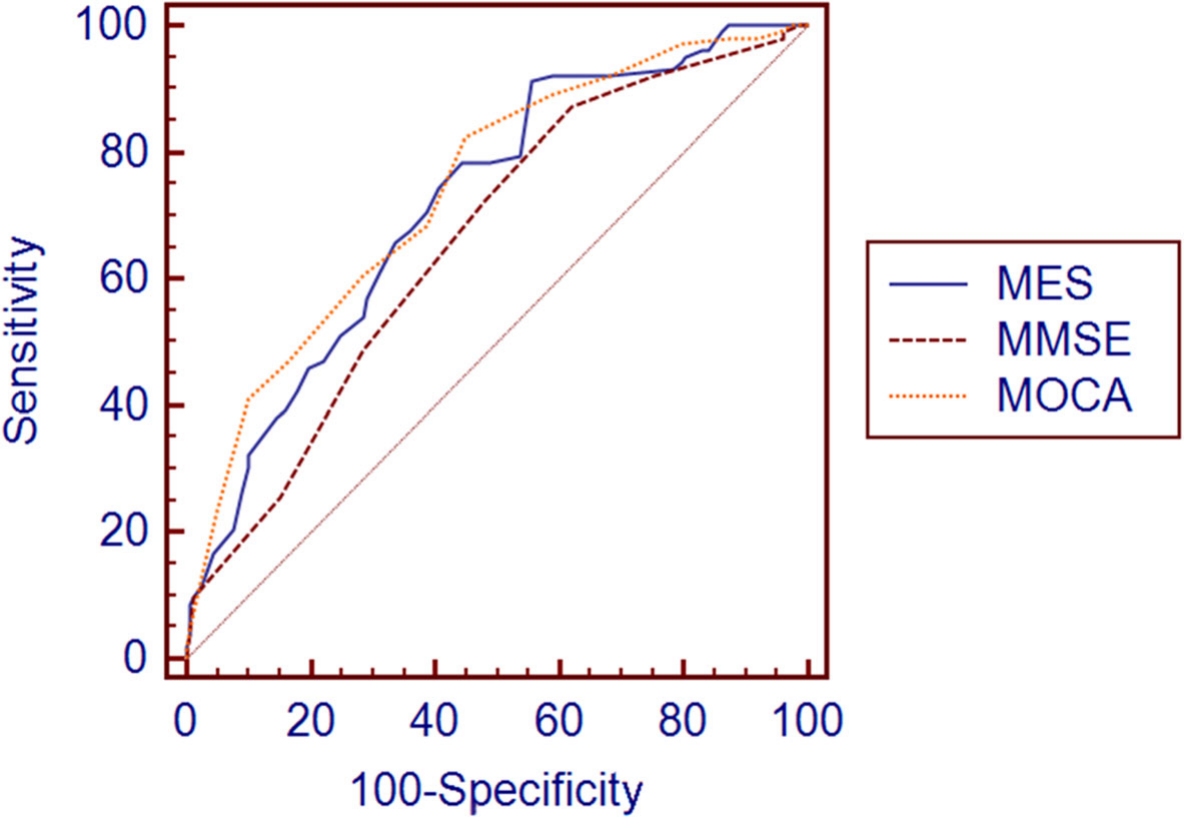
ROC curve for SCD vs MCI

ROC Analyses were used to evaluate the ability of MMSE, MoCA-CV and MES in discriminating individuals among NC, SCD and MCI. As shown in Table 3, the optimal cutoff scores of MMSE, MoCA-CV and MES for discriminating individuals with SCD from NC were determined. The most appropriate MES cutoff was 84, with a sensitivity of 74.3%, a specificity of 60.8% and 0.738 for AUC (95%CI, 0.675–0.801). In comparison, the cutoff scores of MMSE and MOCA-CV (28 and 24, respectively) had poor sensitivity (67.4 and 70.8%, respectively) and specificity (51.0 and 52.9%, respectively), and with smaller AUCs (0.643 and 0.644, respectively) than the MES. Significant differences in the comparison of ROC curves (*P* = 0.0157 for MES and MMSE, *P* = 0.0134 for MES and MoCA-CV) indicated that the MES had a better ability than MMSE and MOCA-CV to detect SCD from NC (Fig. 1). As screening tests, MES, MoCA-CV and MMSE showed a similar trend and more efficient in differentiating MCI from NC (0.886, 0.834, 0.774 respectively for AUCs, Fig. 2). In the ROC analyses for differentiating MCI from SCD (Fig. 3), the MoCA-CV and MES had larger AUCs (0.742 for MoCA-CV, 0.719 for MES) than the MMSE (0.661), no significant difference was found between the AUC of MoCA-CV and MES (Z = 0.625, *P* = 0.5317).

**Table 3:** ROC analyses for MMSE, MoCA-CV and MES to differentiate SCD from NC

Index	AUC	95% Confidence Interval	Cut-off	Sensitivity (%)	Specificity (%)
MMSE	0.643	0.574–0.712	28	67.4	51.0
MoCA-CV	0.644	0.575–0.712	24	70.8	52.9
MES	0.738	0.675–0.801	84	74.3	60.8

*AUC* area under the curve

## Discussion

In this study, the abilities of three cognitive screening tests in identifying individuals with SCD were examined. As a result, MMSE and MoCA-CV were not ideal for the discrimination between SCD and NC. However, MES showed relatively efficacious in identifying SCD from NC, and the optimal cutoff score was 84. We think that the following factors contributed to this result. Firstly, the total score of MES is 100, while the score of MMSE and MOCA-CV is 30. The larger the score range achieved between the group of NC and SCD, the greater the discrimination obtained between them. Secondly, in the comparison of standardized neuropsychological tests between SCD and NC, SCD showed significantly decline only in memory tests. This is corresponding with previous findings that episodic memory decline was seen as one of the earliest markers in the progression to AD [33, 34]. As screening tests, the memory score of MES is 50 in a total of 100, while the MMSE is 6 in a total of 30 and the MOCA-CV is 5 in a total of 30. This significant difference in memory scores in the proportion of total score may also contribute to the ability in detecting SCD. Thirdly, a previous study showed that episodic memory, psychomotor speed and language ability were especially vulnerable in individuals with subjective cognitive decline [35]. The cognitive domains reflected by the subtests of MES are similar to the six indexes in the operational measurements proposed by Jak and Bondi to diagnose MCI and SCD, including instant and delayed memory function, language ability and attention/executive function. Thus, MES is more likely to discriminate SCD from NC in participants with early cognitive decline. Though, MMSE and MoCA-CV are more representing the global cognitive function and have less sensitivity in detecting early cognitive decline.

In the ROC analyses for differentiating MCI from NC, the MES also showed better ability than MMSE and MoCA-CV. On the other hand, these three tests all had larger AUCs compared with their performance on SCD. This is corresponding with the scores obtained by the group of SCD and MCI, and further reflected the progressive cognitive decline from SCD to MCI. In the ROC analyses for differentiating between SCD and MCI, MoCA-CV got a larger AUC compared with MES but no significant difference was found. Associating the abilities of these two tests in identifying SCD from NC, we can infer that MoCA-CV is superior in identifying MCI than SCD, though MES has stable performance in identifying both SCD and MCI.

The pathophysiological process of AD, the most common cause of dementia, is slow and with a long preclinical stage before the appearance of objective cognitive decline [36, 37]. By now, no effective therapy was found in patients with AD dementia. Therefore, screening out the preclinical stages of AD patients without obvious cognitive decline was imperative. In the criteria for preclinical AD published by the National Institute on Aging and the Alzheimer’s Association (NIA-AA) [5], SCD was defined as evidence of subtle change from a baseline level of cognition, poor performance on more challenging cognitive tests, and does not yet meet the criteria for MCI. According to the amyloid cascade hypothesis [38], three stages were proposed by NIA-AA in the development of preclinical AD, and SCD was viewed as the last marker to be affected [5]. However, the hypothesis of the amyloid cascade was increasingly questioned [39]. A study on the cohorts of AD Neuroimaging Initiative (ADNI) showed that Individuals with subtle cognitive decline but no evidence of neuronal injury biomarkers also had a relatively high rate of progression to AD [40]. SCD was considered as an independent risk factor rather than a later marker for progression to MCI and AD [11]. Furthermore, several studies showed that cognitive decline, especially reflected by the sensitive memory tests, were superior to most biomarkers to a certain extent in predicting the development of MCI and AD [33, 34]. In summary, making use of a brief and efficacious neuropsychological test to identify individuals with SCD is important for the prevention and early treatment of MCI and AD. As a screening test, MES showed relatively high sensitivity and specificity in screening SCD from NC in our study.

Something else should be mentioned. As a developing country, China still has a certain proportion of low educated population. Unlike MMSE and MoCA-CV, MES does not require the participants to read and write. Thus, the score of MES is independent of education [18] and it can be applied to the individuals regardless of the educational level of them.

Several limitations should be noted in this study. First, this was a cross-sectional study from a monocentric memory clinic, though measuring the change of cognition overtime should be more accurate than any once measurement. Second, cognitive performance is generally considered to be influenced by age and education level. Due to the sample size, no grouping analysis was performed according to the differences in age and education level. Third, biomarkers associated with AD, such as concentrations of amyloid-β, tau, hyperphosphorylated tau and Apolipoprotein E genotype were not tested in this study. Follow-up studies and detecting biomarkers associated with AD are clearly needed to testify the screening ability of MES in the future.

## Conclusions

Our study found that within standardized neuropsychological tests, the group of subtle cognitive decline showed significant decline only in memory tests compared with the normal controls. As a screening test, MES is more reliable in identifying SCD from normal controls than MMSE and MoCA-CV. The results in this study also suggested that screening scales giving extra weight on the tests of episodic memory, executive function and language function may do better in identifying subtle cognitive decline.

## Data Availability

The datasets used and/or analyzed during the current study are available from the corresponding author on reasonable request.

## Abbreviations

ACE-R: Addenbrooke’s Cognitive Examination Revised
AD: Alzheimer’s disease
ADL: Activities of Daily Living
ADNI: AD Neuroimaging Initiative
AFT: Animal Verbal Fluency Test
AVLT: Auditory Verbal Learning Test
BNT: Boston Naming Test
CDR: Clinical Dementia Rating
CFT: Complex Figure Test
DOT: Digit Ordering Test
FAQ: Functional Activities Questionnaire
MCI: mild cognitive impairment
MES: Memory and Executive Screening
MMSE: Mini-Mental State Examination
MoCA-CV: Montreal Cognitive Assessment-Chinese Version
NC: cognitively normal controls
NIA-AA: the National Institute on Aging and Alzheimer’s Association
PrM: Prospective Memory Test
ROC: Receiver operating characteristic
RPR: rapid plasma regain
SCD: Subtle cognitive decline
SCWT: Stroop Color-Word Test
SDMT: Symbol Digit Modalities Test
TMT: Trail Making Test
TPPA: treponema pallidum particle agglutination

## References

[1] Prince M, Bryce R, Albanese E, Wimo A, Ribeiro W, Ferri CP (2013). The global prevalence of dementia: a systematic review and metaanalysis. Alzheimers Dement.

[2] Albert MS, DeKosky ST, Dickson D, Dubois B, Feldman HH, Fox NC (2011). The diagnosis of mild cognitive impairment due to Alzheimer's disease: recommendations from the National Institute on Aging-Alzheimer's Association workgroups on diagnostic guidelines for Alzheimer's disease. Alzheimers Dement.

[3] Vellas B, Aisen PS, Sampaio C, Carrillo M, Scheltens P, Scherrer B (2011). Prevention trials in Alzheimer's disease: an EU-US task force report. Prog Neurobiol.

[4] Sperling RA, Jack CR, Aisen PS (2011). Testing the right target and right drug at the right stage. Sci Transl Med.

[5] Sperling RA, Aisen PS, Beckett LA, Bennett DA, Craft S, Fagan AM (2011). Toward defining the preclinical stages of Alzheimer's disease: recommendations from the National Institute on Aging-Alzheimer's Association workgroups on diagnostic guidelines for Alzheimer's disease. Alzheimers Dement.

[6] Scarmeas N, Albert SM, Manly JJ, Stern Y (2006). Education and rates of cognitive decline in incident Alzheimer's disease. J Neurol Neurosurg Psychiatry.

[7] Caffo AO, Lopez A, Spano G, Saracino G, Stasolla F, Ciriello G (2016). The role of pre-morbid intelligence and cognitive reserve in predicting cognitive efficiency in a sample of Italian elderly. Aging Clin Exp Res.

[8] Ferreira D, Machado A, Molina Y, Nieto A, Correia R, Westman E (2017). Cognitive variability during middle-age: possible association with Neurodegeneration and cognitive reserve. Front Aging Neurosci.

[9] Jak AJ, Bondi MW, Delano-Wood L, Wierenga C, Corey-Bloom J, Salmon DP (2009). Quantification of five neuropsychological approaches to defining mild cognitive impairment. Am J Geriatr Psychiatry.

[10] Bondi MW, Edmonds EC, Jak AJ, Clark LR, Delano-Wood L, McDonald CR (2014). Neuropsychological criteria for mild cognitive impairment improves diagnostic precision, biomarker associations, and progression rates. J Alzheimers Dis.

[11] Edmonds EC, Delano-Wood L, Galasko DR, Salmon DP, Bondi MW (2015). Alzheimer's Disease Neuroimaging I. Subtle Cognitive Decline and Biomarker Staging in Preclinical Alzheimer's Disease. J Alzheimers Dis.

[12] Guo QHLC, Hong Z (2001). Reliability and validity of auditory verbal learning test on Chinese elderly patients. J Chin Ment Health.

[13] Zhao QHGQ, Shi WX, Zhou Y, Hong Z (2007). Category verbal fluency test in identification and differential diagnosis of dementia. Chin J Clin Psychol.

[14] Guo QHHZ, Shi WX, Sun YM, Lv CZ (2006). Boston naming test using by Chinese elderly, patient with mild cognitive impairment and Alzheimer’s dementia. J Chin Ment Health.

[15] Zhao Q, Guo Q, Li F, Zhou Y, Wang B, Hong Z (2013). The Shape Trail Test: application of a new variant of the Trail making test. PLoS One.

[16] Mioshi E, Dawson K, Mitchell J, Arnold R, Hodges JR (2006). The Addenbrooke's cognitive examination revised (ACE-R): a brief cognitive test battery for dementia screening. Int J Geriatr Psychiatry.

[17] Nasreddine ZS, Phillips NA, Bedirian V, Charbonneau S, Whitehead V, Collin I (2005). The Montreal cognitive assessment, MoCA: a brief screening tool for mild cognitive impairment. J Am Geriatr Soc.

[18] Guo QH, Zhou B, Zhao QH, Wang B, Hong Z (2012). Memory and executive screening (MES): a brief cognitive test for detecting mild cognitive impairment. BMC Neurol.

[19] De Roeck EE, De Deyn PP, Dierckx E, Engelborghs S (2019). Brief cognitive screening instruments for early detection of Alzheimer's disease: a systematic review. Alzheimers Res Ther.

[20] Bosco A, Spano G, Caffo AO, Lopez A, Grattagliano I, Saracino G (2017). Italians do it worse. Montreal Cognitive Assessment (MoCA) optimal cut-off scores for people with probable Alzheimer's disease and with probable cognitive impairment. Aging Clin Exp Res.

[21] Katzman R, Zhang MY, Ouang Ya Q, Wang ZY, Liu WT, Yu E (1988). A Chinese version of the mini-mental state examination; impact of illiteracy in a Shanghai dementia survey. J Clin Epidemiol.

[22] Juva K, Sulkava R, Erkinjuntti T, Ylikoski R, Valvanne J, Tilvis R (1995). Usefulness of the clinical dementia rating scale in screening for dementia. Int Psychogeriatr.

[23] Staff PH (1980). ADL-assessment. Scand J Rehabil Med Suppl.

[24] Hamilton M (1960). A rating scale for depression. J Neurol Neurosurg Psychiatry.

[25] McKhann GM, Knopman DS, Chertkow H, Hyman BT, Jack CR, Kawas CH (2011). The diagnosis of dementia due to Alzheimer's disease: recommendations from the National Institute on Aging-Alzheimer's Association workgroups on diagnostic guidelines for Alzheimer's disease. Alzheimers Dement.

[26] Lu J, Li D, Li F, Zhou A, Wang F, Zuo X (2011). Montreal cognitive assessment in detecting cognitive impairment in Chinese elderly individuals: a population-based study. J Geriatr Psychiatry Neurol.

[27] Livner A, Laukka EJ, Karlsson S, Backman L (2009). Prospective and retrospective memory in Alzheimer's disease and vascular dementia: similar patterns of impairment. J Neurol Sci.

[28] Fellows Robert P, Schmitter-Edgecombe Maureen (2019). Symbol Digit Modalities Test: Regression-Based Normative Data and Clinical Utility. Archives of Clinical Neuropsychology.

[29] Hoppe CD, Muller UD, Werheid KD, Thone AD, von Cramon YD (2000). Digit ordering test: clinical, psychometric, and experimental evaluation of a verbal working memory test. Clin Neuropsychol.

[30] Guo QHHZ, Lv CZ, Zhou Y, Lu JC, Ding D (2005). Application of Stroop color-word test on Chinese elderly patients with mild cognitive impairment and mild Alzheimer’s dementia. Chin J Neuromed.

[31] Guo QHLC, Hong Z (2000). Application of Rey-Osterrieth complex figure test in Chinese normal old people. J Chin Clin Psychol.

[32] Teng E, Becker BW, Woo E, Knopman DS, Cummings JL, Lu PH (2010). Utility of the functional activities questionnaire for distinguishing mild cognitive impairment from very mild Alzheimer disease. Alzheimer Dis Assoc Disord.

[33] Jedynak BM, Lang A, Liu B, Katz E, Zhang Y, Wyman BT (2012). A computational neurodegenerative disease progression score: method and results with the Alzheimer's disease neuroimaging initiative cohort. Neuroimage.

[34] Gomar JJ, Bobes-Bascaran MT, Conejero-Goldberg C, Davies P, Goldberg TE (2011). Alzheimer's Disease Neuroimaging I. Utility of combinations of biomarkers, cognitive markers, and risk factors to predict conversion from mild cognitive impairment to Alzheimer disease in patients in the Alzheimer's disease neuroimaging initiative. Arch Gen Psychiatry.

[35] Kielb S, Rogalski E, Weintraub S, Rademaker A (2017). Objective features of subjective cognitive decline in a United States national database. Alzheimers Dement.

[36] Villemagne VL, Burnham S, Bourgeat P, Brown B, Ellis KA, Salvado O (2013). Amyloid beta deposition, neurodegeneration, and cognitive decline in sporadic Alzheimer's disease: a prospective cohort study. Lancet Neurol.

[37] Morris JC (2005). Early-stage and preclinical Alzheimer disease. Alzheimer Dis Assoc Disord.

[38] Jack CR, Knopman DS, Jagust WJ, Shaw LM, Aisen PS, Weiner MW (2010). Hypothetical model of dynamic biomarkers of the Alzheimer's pathological cascade. Lancet Neurol.

[39] Drachman DA (2014). The amyloid hypothesis, time to move on: amyloid is the downstream result, not cause, of Alzheimer's disease. Alzheimers Dement.

[40] Toledo JB, Weiner MW, Wolk DA, Da X, Chen K, Arnold SE (2014). Neuronal injury biomarkers and prognosis in ADNI subjects with normal cognition. Acta Neuropathol Commun.

